# Spatially invariant computations in stereoscopic vision

**DOI:** 10.3389/fncom.2012.00047

**Published:** 2012-07-16

**Authors:** Michel Vidal-Naquet, Sergei Gepshtein

**Affiliations:** ^1^Brain Science Institute, RIKEN, Wako-shiSaitama, Japan; ^2^The Salk Institute for Biological StudiesLa Jolla, CA, USA

**Keywords:** adaptive, binocular matching, complex cell, correlation, flexible matching, perception of slant, stereopsis

## Abstract

Perception of stereoscopic depth requires that visual systems solve a correspondence problem: find parts of the left-eye view of the visual scene that correspond to parts of the right-eye view. The standard model of binocular matching implies that similarity of left and right images is computed by inter-ocular correlation. But the left and right images of the same object are normally distorted relative to one another by the binocular projection, in particular when slanted surfaces are viewed from close distance. Correlation often fails to detect correct correspondences between such image parts. We investigate a measure of inter-ocular similarity that takes advantage of spatially invariant computations similar to the computations performed by complex cells in biological visual systems. This measure tolerates distortions of corresponding image parts and yields excellent performance over a much larger range of surface slants than the standard model. The results suggest that, rather than serving as disparity detectors, multiple binocular complex cells take part in the computation of inter-ocular similarity, and that visual systems are likely to postpone commitment to particular binocular disparities until later stages in the visual process.

## Introduction

**Figure 1:**
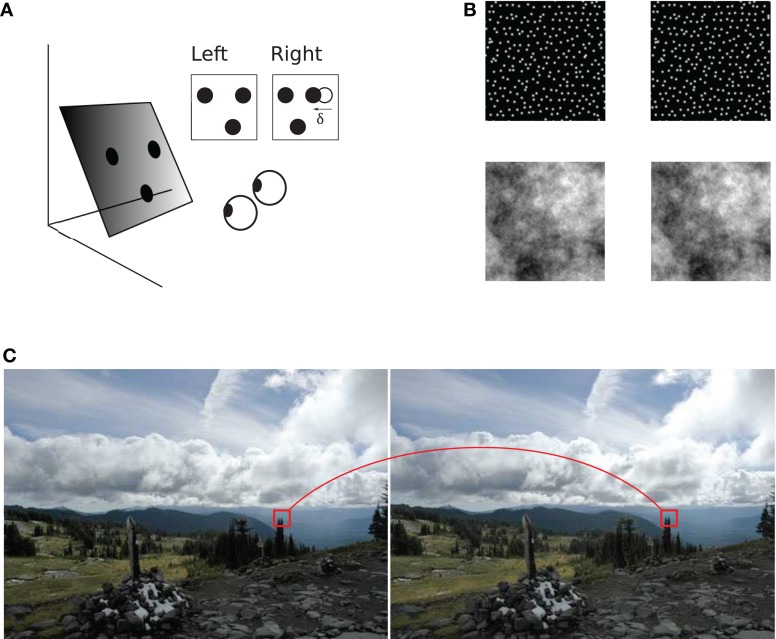
**Binocular geometry. (A)** A slanted plane with three dots painted on it is viewed from two slightly different vantage points. Left and right projections of the dots are shown in the insets. Coordinates of dot projections in the two images are generally different, illustrated for one of the dots using vector δ. The horizontal extent of this vector is called horizontal binocular disparity. The triangle formed by the three dots in the right image is distorted with respect to the triangle in the left image **(B)** Examples of stereograms (image pairs) used in the present study: a random-dot stereogram on the top and a stereogram with 1/f luminance distribution on the bottom. Both stereograms depict a slanted plane which the reader may experience by cross fusion. **(C)** Binocular correspondence. The visual system must establish which parts of the two images correspond to the same source in the scene. A pair of such corresponding image parts is shown in a stereogram of a natural scene (“Accidental stereo pair.” Online image. Flickr. http://www.flickr.com/photos/abulafia/829612/, Creative Commons).

Stereoscopic vision depends on *binocular matching*: a process that finds which parts of the left and right eye's images correspond to the same source in the visual scene (Figure 1). The difference between positions of the corresponding image parts is called *binocular disparity*, a key source of information for perception of stereoscopic depth.

In the standard view of binocular matching, the corresponding parts of left and right images are found using *inter-ocular correlation* as the measure of image similarity. This view is supported by neurophysiological evidence. The *disparity energy model* (Ohzawa et al., 1990; Qiang, 1994; Cumming and Parker, 1997; Ohzawa, 1998; Cumming and DeAngelis, 2001; Haefner and Cumming, 2008) describes function of binocular complex cells which are thought to play a key role in the computation of binocular disparity (and which are sometimes described as “disparity detectors”). Responses of modeled binocular complex cells to some stimuli are well approximated by a computation similar to inter-ocular correlation (Fleet et al., 1996; Qian and Zhu, 1997; Anzai et al., 1999), and so a simplifying assumption is often made that inter-ocular correlation can be used to predict outcomes of the computation of similarity in biological vision. In psychophysical studies of stereopsis, for example, inter-ocular correlation is commonly used to explain limitations of stereoscopic vision (Tyler, 1973; Cormack et al., 1991; Banks et al., 2004, 2005; Filippini and Banks, 2009), in particular the decline in the ability for stereopsis at large slants of stimulus surfaces.

The computation of similarity of left and right images using inter-ocular correlation has two shortcomings. First, correlation of image regions fails to capture an important characteristic of complex cells: spatial invariance of their responses (even though the disparity energy model does capture this invariance). The disregard for spatial invariance misses an important aspect of the biological computation. Studies of other visual functions showed that spatial invariance endows visual systems with important computational abilities, e.g., in object perception (Riesenhuber and Poggio, 1999; Ullman et al., 2002; Yu et al., 2002; Serre et al., 2007a,b) and in efficient encoding of natural scenes (Hyvarinen and Hoyer, 2000; Karklin and Lewicki, 2009).

Second, inter-ocular correlation is biased in favor of stimuli that are uncommon in the natural viewing conditions. Inter-ocular correlation is “rigid” in the sense it does not tolerate large distortions of corresponding image parts: correlation peaks where image parts are identical and it rapidly declines where image parts are dissimilar. But only rarely do identical left and right images occur in the natural environment. Because of the geometry of binocular projection, parts of the left and right images are generally dissimilar (Figure 1A), especially when stimulus surfaces are slanted and viewed from a short distance (Pollard et al., 1986; Filippini and Banks, 2009). It is therefore not surprising that a recent study of human perception found that the correlation operation fails to explain human perception in stimuli that involve slanted surfaces (Allenmark and Read, 2010). We refer to this implicit bias of matching by correlation as the *assumption of uniform disparity*.

In the following we propose that the computation of binocular similarity in biological vision should be modeled using an operation which, first, takes advantage of the spatial invariance found in binocular complex cells and, second, avoids the inapt assumption of uniform disparity. We investigate a “flexible” measure of similarity that tolerates distortions of the corresponding parts of left and right images. We implement this measure using a *MAX-pooling operation*, which has been successfully used for modeling spatially invariant computations by complex cells in service of other functions of biological vision (Riesenhuber and Poggio, 1999; Serre et al., 2007a,b).

In a series of computational experiments, we simulate a tilt discrimination task using stimuli that portray a wide range of surface slants. The stimuli are composed of two types of texture: random dots (common in psychophysical studies of stereopsis, e.g., Banks et al., 2004; Filippini and Banks, 2009; Allenmark and Read, 2010) and patterns that imitate statistics of luminance in natural images (Ruderman and Bialek, 1994).

We find that the spatially invariant computation of inter-ocular similarity supports excellent performance over a significantly larger range of stimulus slants than the rigid computation. This is because the flexible measure of similarity can adapt to different amounts of inter-ocular distortion in different parts of the stimulus.

We also find that in stimuli with naturalistic image statistics, the flexible measure is more effective than methods previously advanced to overcome inter-ocular distortions, such as image blurring, supporting the view that spatially invariant computation of inter-ocular similarity is particularly suitable for stereoscopic vision in the natural visual environment.

## Models and methods

**Figure 2:**
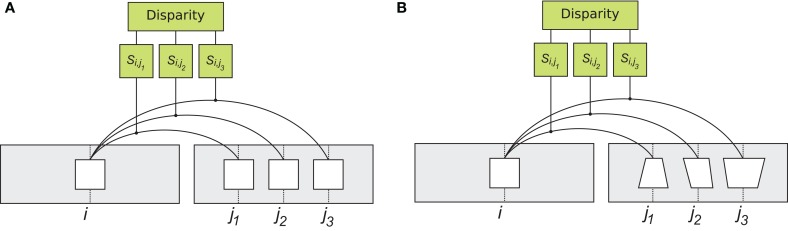
**Rigid and flexible matching.** To determine corresponding patches, the system computes visual similarity between a left image patch (“template”) at location *i* and all the patches on the epipolar line of the right image, at locations *j*_1_, *j*_2_ and so on (only three right image patches are shown). Right image patch with largest similarity *S*_*i*,*j*_ is considered the corresponding patch. **(A)** matching with the rigid similarity measure. A patch from the left image, is compared to patches in the right image, using a correlation type operator. The operation is rigid in the sense that patches are compared in a pixel wise manner (Equation 1). **(B)** matching with the flexible similarity matching. The flexible matching allows to match corresponding patches that are distorted due to viewing geometry. This is achieved by searching through a space of possible distortions and finding the particular distortion that best suits the “template”. The implementation involves spatial invariant computational units, illustrated in detail in Figure 3.

We first describe the two methods for measurement of inter-ocular similarity compared in our experiments: rigid matching and flexible matching (Figure 2). We then describe the computations we used to evaluate performance of these matching methods. (We chose to do so using a tilt discrimination task because it allowed us to compare matching methods comprehensively: across many directions of disparity change, which is particularly important in the complex stimulus of Experiment 2.)

### Rigid matching

Normalized Cross-correlation is commonly used for modeling of binocular matching in biological vision (Tyler and Julesz, 1978; Cormack et al., 1991; Banks et al., 2004, 2005; Filippini and Banks, 2009). For image parts (“patches” or “templates”) **L** from the left image and **R** from the right image, this measure is

(1) $$\begin{array}{c} \text{C(L, R)}=\frac{1}{\sigma_{L}\sigma_{R}}\sum_{x,y=1}^{N}(L_{\left(x,y\right)}-\bar{L})(R_{\left(x,y\right)}-\bar{R}), \end{array}$$

where *L*_(*x*,*y*)_ and *R*_(*x*,*y*)_ are the luminances at coordinates (*x*, *y*), $\bar{L}$ and $\bar{R}$ are the average luminances, σ_*L*_ and σ_*R*_ are the standard deviations of luminance distributions, and *N* is the number of image elements within each patch used in the computation.

**Figure 3:**
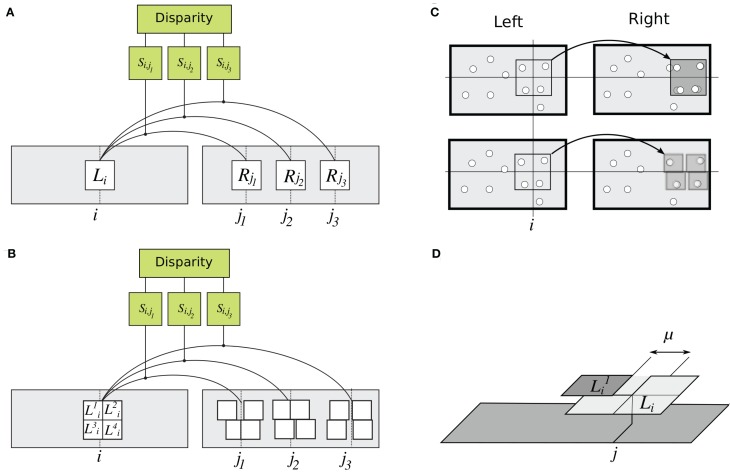
**Details of rigid and flexible matching. (A)** Rigid matching. Patch *L*_*i*_ at horizontal position *i* in the left image is compared to several patches in the right image, at positions *j*_1_, *j*_2_, and *j*_3_. For each pair of left and right patches, similarity is computed as in Equation 1, yielding similarity measures *S*_*i*,*j*_1__, *S*_*i*,*j*_2__, and *S*_*i*,*j*_3__. A solution to the correspondence problem is the pair of patches for which similarity is the highest. **(B)** Implementation of flexible matching. Template *L*_*i*_ in the left image is divided to four sub-templates *L*^*k*^_*i*_, *k* ∈ [1,… 4]. Binocular similarity is computed for each sub-template over a small range of horizontal locations (explained in panel D). This way, highest similarity is found separately for each sub-template, illustrated here by different displacements of the sub-templates. **(C)** Illustrations of rigid and flexible matching. *Top:* Rigid matching. A left-image patch (a “template”) is superimposed on the corresponding right-image patch. Image features (represented by white disks) in the left and right patches are not aligned, yielding low correlation between the patches (Equation 1). *Bottom:* Flexible matching. Now the left-image patch (“template”) is divided to parts (“sub-templates”) which can “move” independent of one another and thus warp the basis template. The warping enables good registration of image features despite distortions induced by binocular projections. **(D)** Parameters of flexible matching. In this example, similarity of template *L*_*i*_ at location *j* of the right image is computed using flexible matching. Template *L*_*i*_ is divided to four sub-templates *L*^*k*^_*i*_, *k* ∈ [1,… 4]. Correlation values are computed for sub-template *L*^*k*^_*i*_ over set of contiguous locations **M**^*j*^_*k*_. **M**^*j*^_*k*_ is shown for one sub-template (*k* = 1), indicated by the double arrow. Size μ of **M**^*j*^_*k*_ is called *template flexibility*. Computing correlation of *L*^*k*^_*i*_ over locations **M**^*j*^_*k*_ in the right image, and finding the maximal value, yields the sub-template similarity *S*^*k*^_*i*,*j*_ (MAX-pooling operation, Equation 3). This process is repeated for each sub-template. The maximal correlation of the four sub-templates are averaged to obtain the measure of similarity of template *L*_*i*_ at location *j* in the right image (Equation 5).

This measure is “rigid” in the sense the inter-ocular similarity is computed using unaltered image patches, i.e., as they are in the left and right projections of the visual scene (Figures 2A and 3A). The rigid computation of similarity favors matching of image parts that are identical (up to a luminance multiplication and shift), which is why estimates of similarity of corresponding patches rapidly decline when luminance patterns in the left and right images are misaligned (Figure 3C, top). Thus, rigid matching is likely to miss binocular correspondences when local image distortions are large, which happens when surface slant is high.

To contrast the rigid measure of inter-ocular similarity with the measure we review next (Equation 5), we write it as

(2) $$\begin{array}{c} S_{i,j}^{\text{rig}}={\text{C(L}}_{\text{i}},{\text{R}}_{\text{j}}), \end{array}$$

where C is as in Equation 1, and *L*_*i*_ and *R*_*j*_ stand for the left and right image patches of the same size.

### Flexible matching

We compared the rigid measure of inter-ocular similarity with another measure, introduced here, which we called “flexible” because it tolerates small distortions of corresponding image parts. Now the computation of Equation 1 is applied independently to parts (“sub-patches” or “sub-templates”) of **L** and **R**. The parts may undergo small independent displacements with respect to their original locations, emulating properties of multiple complex cells tuned to adjacent spatial locations (Riesenhuber and Poggio, 1999; Ullman et al., 2002; Serre et al., 2007a,b; Ullman, 2007).

Flexible matching is illustrated in Figures 3B–D. Patch **L**_*i*_ is divided to *T* parts: sub-templates **L**^*k*^_*i*_, where *k*∈[1, …, *T*] is the sub-template index. (In the experiments we tested divisions of the templates into different numbers of sub-templates of equal size: four, nine, and 16.) Patch similarity *S*^flex^_*i*,*j*_ is computed in two steps:

1. Correlation is determined as in Equation 1 separately for each sub-template **L**^*k*^_*i*_, over a set of contiguous horizontal coordinates **M**^*j*^_*k*_ (Figures 3C–D). The maximal similarity is
  (3) $$\begin{array}{c} S_{i,j}^{k}=\underset{u\in {\text{M}}_{k}^{j}}{\max}\left({\text{C(L}}_{i}^{k}{\text{, R}}_{u}^{k})\right), \end{array}$$
  where *u* is the horizontal position of sub-template in the right image. Equation 3 is the MAX-pooling operation. Length μ of set **M**^*j*^_*k*_ is called *template flexibility*. It is a range of locations near location *j* in the right image, for which sub-template similarities are computed, such that
  (4) $$\begin{array}{c} \mu =\max\left({\text{M}}_{k}^{j}\right)-\min\left({\text{M}}_{k}^{j}\right)+1. \end{array}$$
  Template flexibility determines the range of inter-ocular distortions tolerated by the matching procedure. (In these experiments, all sub-templates had the same flexibility μ.)
2. Results of MAX-pooling are combined across sub-templates:
  (5) $$\begin{array}{c} S_{i,j}^{\text{flex}}=\frac{1}{T}\sum_{k=1}^{T}S_{i,j}^{K}. \end{array}$$
  This way, best match is found for each sub-template—over a small image vicinity, independent of other sub-templates, and without computing disparities for each sub-template—possibly “warping” the template. The maximal amount of warping depends on template flexibility μ. (As explained in section *Computation of tilt* below, visual systems may automatically select the magnitude of μ that is most suitable for the local slant in the stimulus.)

### Computation of disparity

In both rigid and flexible methods, inter-ocular correspondences are found by computing similarity (*S*) between multiple parts of the left and right images of the scene (Figures 1, 2). Suppose a small part of the left image, centered on location *i*, is compared to multiple parts of the right image, at locations *j* (Figure 3A). (For simplicity, we consider only image parts at the same height in the two images, i.e., we assume the *epipolar constraint*; Hartley and Zisserman, 2003). Thus, *S*_*i*,*j*_ is the similarity between image patches at locations *i* and *j*, in the left and right images, respectively. The patch at j^*^ that is most similar to the patch at *i* is a solution to the correspondence problem:

(6) $$\begin{array}{c} j^{*}=\text{arg }\underset{j}{\max}\;S_{i,j}, \end{array}$$

such that the estimated binocular disparity at *i* is

(7) $$\begin{array}{c} \delta_{i}=j^{*}-i. \end{array}$$

### Computation of tilt

We compared how efficiently the rigid and flexible matching methods estimated inter-ocular similarity using a winner-take-all (WTA) computation (which is believed to be widely implemented in cortical circuits, e.g., Abeles, 1991; Sakurai, 1996; Lee et al., 1999; Flash and Sejnowski, 2001). Assuming that different magnitudes of template flexibility μ correspond to different sizes of respective fields in complex cells, the WTA computation amounts to the competition between complex cells with respective fields of different sizes.

**Figure 4:**
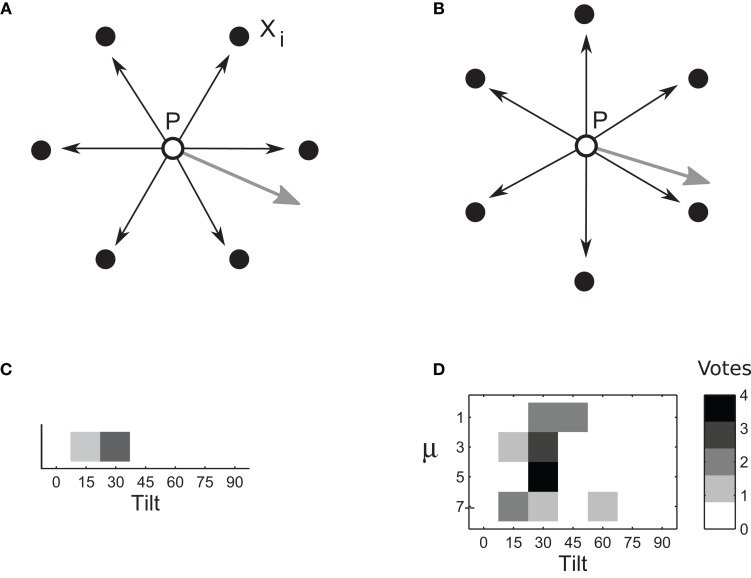
**Computation of tilt. (A–B)** Surface tilt at point *P* in the left image is computed using disparities δ_*i*_, *i*∈ [1,… 6] estimated for six sampling points: vertices of a hexagon centered on *P*. (Different sets of sampling points are shown in **A** and **B**. Four such sets were used for computation of one tilt.) Surface gradient (gray arrows) is proportional to weighted vector sum $\sum \delta_{i}\;\vec{PX_{i}}$. The computation of tilt was repeated several times, using a different set of samplings points every time, all centered on *P*. **(C)** Each set of sampling points provides one vote for tilt estimate. Tilt estimates from multiple sampling sets are combined in a vector whose entries represent numbers of votes for each particular tilt. Intensities of cells in this figure represent the number of votes. The tilt that receives most votes wins. **(D)** An example of voting matrix used in the adaptive-flexible approach. The process described in Figures 4A–C is repeated for different magnitudes of template flexibility μ (four magnitudes are used in this illustration), yielding multiple estimate vectors concatenated in a voting matrix. The row that corresponds to the most suitable μ is likely to have most consistent votes. Accordingly, the cell with a largest number of votes is selected. (Here, it is the μ of 5 and the tilt of 30.)

We simulated estimation of tilt at point *P* in the left image using several samples of disparity δ_*i*_: six points *X*_*i*_ forming vertices of a regular hexagon centered on *P* (Figure 4A). Disparities δ_*i*_ were computed as in Equations 6–7 for each sampling point. The similarity measure of Equation 6 was implemented separately for each matching method—rigid matching, flexible matching with fixed μ, and flexible matching with variable, “adaptive” μ—each leading to a separate estimate of tilt, as follows.

We took advantage of the fact that the sum of vectors $\vec{PX_{i}}$, weighted by disparities δ_*i*_:

(8) $$\begin{array}{c} g=\sum \delta_{i}\;\vec{PX_{i}}, \end{array}$$

is proportional to surface gradient at *P*. Tilt θ at point *P*, computed separately for each matching method, therefore is

(9) $$\begin{array}{c} \theta =\text{arctan}\frac{g_{y}}{g_{x}}, \end{array}$$

where **g** = [*g*_*x*_, *g*_*y*_]^T^. The relation between disparity gradient **g**, inter-ocular distance *i*, slant *S*, and viewing distance *d* is (Pollard et al., 1986):

(10) $$\begin{array}{c} \left|g\right|=\frac{I}{d}\text{arctan}(s). \end{array}$$

Final estimates of tilt were derived by way of population vote, in which several sets of sampling points were used to provide independent estimates.

#### Population vote for rigid matching

In rigid matching, tilt at point *P* was estimated using four different sets of sampling points, yielding four tilt estimates. Each set contained six different points, all centered on *P* (Figure 4B). The four estimates were assembled in a one-dimensional *voting matrix*, whose entries were cumulative counts of “votes” supporting a particular tilt (Figure 4C). (In a separate experiment, we determined that performance of the voting method, using several sampling sets, was better than performance based on the same number of sampling points in one large set.)

#### Population vote for flexible matching

In flexible matching, the voting matrix was two-dimensional. Tilt estimates were obtained: for different sampling sets, as in rigid matching, but also for different magnitudes of template flexibility μ (Figure 4D). The different entries in the matrix represented different hypotheses about the tilt. As in rigid matching, the entry with the largest number of votes was taken as the indicator of tilt. Since flexible matching with a fixed magnitude of μ favored a particular range of slants (Figure 7), this procedure found the magnitude of μ that was most useful for the present stimulus.

We summarize the WTA computation in pseudo-code:

1. Initialize a 4 × 7 voting matrix to 0,
2. *For each magnitude of template flexibility* μ (*e.g.,* μ ∈ [1 3 5 7]):
  *For each set of sampling points (four sets of six points each)*:
  - compute disparity (Equations 6–7),
  - compute tilt (Figure 4A),
  - *increment the voting matrix cell that corresponds to the estimated tilt and the magnitude of template flexibility (Figure 4D)*.
3. Select the tilt indicated by the cell with a highest number of votes.

Each cell in the voting matrix contained the number of times a particular tilt was voted for, using particular template flexibility μ (Figure 4D). The winning tilt was the one that received most votes. We call this computation “adaptive” because it selects a magnitude of μ that is most suitable for current stimulation. We refer to computations that use a single magnitude of μ, i.e., where the voting matrix consists of a single row, as “flexible matching with fixed μ”.

We performed two experiments. In Experiment 1, each stimulus represented a planar surface and thus it was characterized by a single tilt (of seven possible tilts), such that a single voting matrix was used for each stimulus (with 28 entries generated by four magnitudes of template flexibility and seven tilts). We also tested larger magnitudes of μ and larger numbers of sub-templates, as described in Results.

In Experiment 2, the stimulus represented a concentric sinusoidal surface whose tilts spanned the range of 0–360°. A voting matrix of 4 × 360 was derived for every location in the stimuli. The resulting matrices were each filtered using a 1 × 20 Gaussian kernel, to ensure additive contribution of the nearby votes.

Notably, the computation of tilt made no commitment to particular magnitudes of template flexibility, and consequently no commitment to particular magnitudes of binocular disparity. Multiple hypotheses about template flexibility and binocular disparity coexisted, yielding a single estimate of tilt.

### Stimuli

Stereoscopic stimuli were generated using two types of luminance patterns and they depicted two types of surfaces.

#### Luminance patterns

Images of stimulus stereograms contained either textures with a 1/f luminance power spectrum or random-dot textures. The former reproduced the scale invariant property of natural scenes (Ruderman and Bialek, 1994). The latter are commonly used in psychophysical and computational studies of stereopsis. In both cases, the image pairs were obtained by first generating a *source image* (random-dot or 1/f) and then displacing pixels by half the disparity signal in opposite directions, to obtain the left and right images (as in Banks et al., 2004). In random-dot sources images, the dots formed a perturbed hexagonal grid of 40 × 40 dots. Dots were displaced from positions in a hexagonal grid in random directions, uniformly in all directions, and for a random distance of up to half of inter-dot distance. The 1/f source images were obtained by first generating a white-noise image, whose Fourier amplitude was then modified to obtain the desired power spectrum. Images of both kinds were 512 × 512 pixels. Left and right images were blurred using a Gaussian kernel of size 6 × 6 pixels and standard deviation of 1.5 pixels, to emulate the effect of the optical point-spread function (Campbell and Gubisch, 1966; Banks et al., 2004).

#### Surfaces

**Figure 5:**
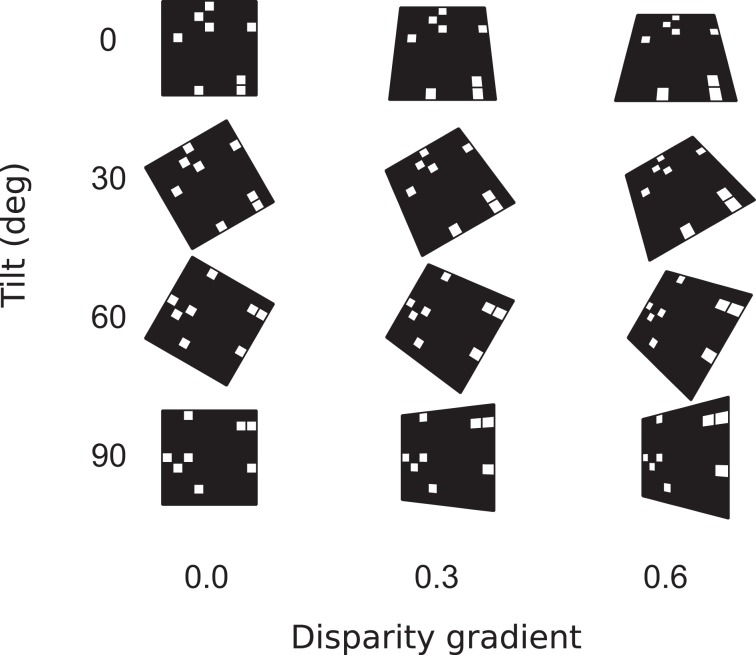
**Surface parameters.** Surface orientation in three dimensions is parameterized by slant and tilt. Slant is the angle between the line of sight and surface normal. In our experiments, surfaces were characterized by their disparity gradients. (The relationship of disparity gradient and slant is explained in Models, Equation 10.) Tilt is the angle between the projection of surface normal on the frontal plane and the (0,*x*) axis in the frontal plane.

In Experiment 1, stimuli depicted flat surfaces at different slants and tilts (Figures 1A,B), using both random-dot and 1/f luminance textures. For each combination of slant and tilt, we generated 100 random-dot stimuli and 100 naturalistic stimuli. The tilts ranged from 0 to 90°, and surface disparity gradients (Equation 10) ranged from 0 to 0.95 (Figure 5). Tilt estimates were derived for stimulus center using Equation 10. For each slant, tilt, and stimulus type (random-dot or 1/f), we computed accuracy of tilt discrimination using the rigid and flexible matching methods. (Accuracy is the frequency of cases where the estimated tilt was equal to the true tilt). Figures 7–8 are summaries of accuracy, plotted as a function of slant for the two matching methods, using different luminance patterns in the stimulus.

**Figure 6:**
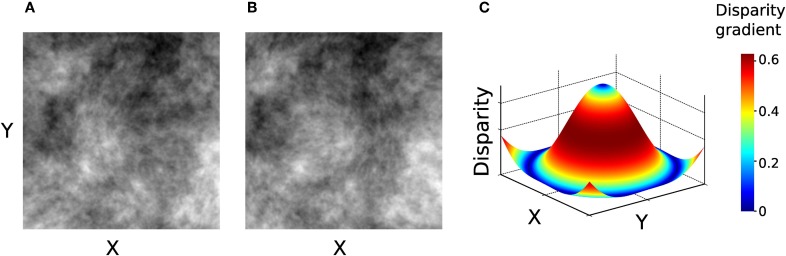
**Stimuli used in Experiment 2. (A–B)** Left and right images of the stimulus. **(C)** Disparity signal encoded in the image pair in panels **A–B**. Surface color represents the magnitude of disparity gradient. This stimulus contains the entire range of tilts (0–359°).

In Experiment 2, the stimuli were generated using only 1/f luminance textures, depicting a surface whose depth was modulated according to a concentric sinusoidal function, illustrated in Figure 6.

The slope of this surface is the disparity gradient. The larger the slope, the stronger the inter-ocular dissimilarity, and so a larger template flexibility is needed to attain accurate binocular matching.

## Results

### Experiment 1

We measured accuracy of tilt estimation as a function of slant using different matching methods:

#### Rigid matching

**Figure 7:**
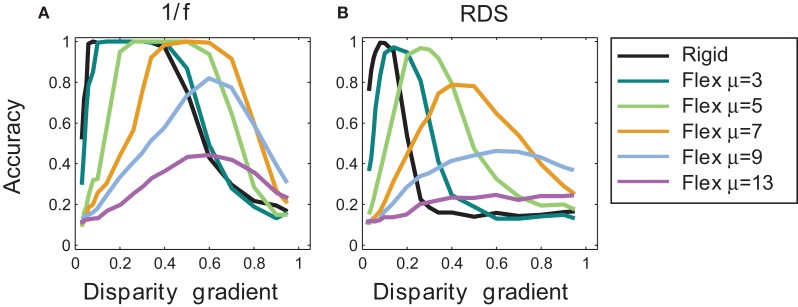
**Tilt discrimination performance in Experiment 1: rigid matching vs. flexible matching with fixed flexibility. (A)** Results for 1/f stimuli, using rigid matching (black curve) and flexible matching with fixed magnitudes of template flexibility μ (colored curves). Accuracy of tilt estimation is plotted as a function of surface slant. (Perfect performance is 1 and random performance is 0.14.) In flexible matching, performance depends on template flexibility μ: the higher the template flexibility, the larger the slant at which performance peaks. **(B)** Results for random-dot stereograms, using the same convention as in panel A.

Outcomes of rigid matching in Experiment 1 are represented by the black curve in Figure 7, for 1/f stimuli in panel A and for random-dot stimuli in panel B. For 1/f stimuli, performance of the rigid procedure peaked at the disparity gradients of 0.1–0.4. For random-dot stimuli, performance peaked near the disparity gradient of 0.16 and then abruptly decreased, falling to half of its peak performance at the disparity gradient of 0.2. For disparity gradients larger than 0.4 in 1/f stimuli, and larger than 0.16 in random-dot stimuli, the inter-ocular distortion of corresponding patches was too large for the rigid procedure to find correct matches, which explains the sharp decrease in performance.

#### Flexible matching with fixed flexibility

Outcomes of flexible matching with fixed magnitudes of μ are represented by the colored curves in Figure 7, for 1/f stimuli in panel A and for random-dot stimuli in panel B. (The black curve represents outcomes of rigid matching.) As template flexibility increased, the peak of performance shifted toward the higher disparity gradients for both 1/f and random-dot stimuli. Maximal performance was high for small and intermediate magnitudes of μ, but it deteriorated at the large magnitudes of μ (9 and 13).

The preference for higher disparity gradients at larger magnitudes of μ is expected because large template flexibility entails high tolerance to dissimilarity of corresponding image patches. But as flexibility μ is increased yet further, the matching is increasingly afflicted by spurious matches, which explains the drop of performance at the two largest magnitudes of μ.

In other words, Figure 7 captures a tradeoff between effects of different magnitudes of template flexibility. Flexible matching with low magnitudes of μ favors matching of similar image patches, making the matching procedure miss the corresponding patches under high inter-ocular deformation at large disparity gradients. Flexible matching with high magnitudes of μ does not miss the correspondences under high inter-ocular deformation, but it is prone to register spurious matches. In effect, performance curves for flexible matching with fixed magnitudes of μ shift along the dimension of disparity gradient: the larger μ the farther the shift toward large disparity gradients.

#### Flexible matching with variable flexibility

**Figure 8:**
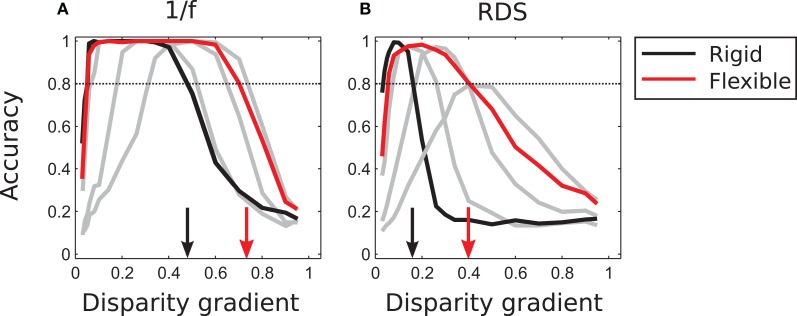
**Tilt discrimination performance in Experiment 1: rigid matching vs. flexible matching with adaptive selection of template flexibility. (A)** Results for 1/f stimuli. Flexible matching with variable template flexibility (red curve) attained a much larger range of correct classification than rigid matching (black curve). (The gray curves represent performance of flexible matching with different fixed magnitudes of template flexibility μ, the same as those rendered as colored curves in Figure 7, but excluding μ of 9 and 13.) **(B)** Results for random-dot stimuli, using the same convention as in panel A. In both panels, the arrows mark the magnitudes of disparity gradient at which the descending arms of performance curves crossed the 0.8 level of accuracy.

As demonstrated in Figure 7, a fixed amount of template flexibility favors a particular range of slants. A system employing different magnitudes of template flexibility would be able to take advantage of the degree of flexibility that is most suitable for current stimulus and thus yield reliable performance for a large range of slants. Performance of such an “adaptive” system (described in section “Population vote for flexible matching” in “Models and Methods”) is represented by the red curve in Figure 8. (The black curve is the same as in Figure 7; it represents outcomes of rigid matching.) For 1/f stimuli, maximal performance of adaptive matching was reached for disparity gradients in the range of 0.1–0.6. For the random-dot stimuli, performance of adaptive matching peaked at the disparity gradient of 0.2. The red curve in Figure 8 effectively circumscribes the pertinent curves of Figure 7. (Very large magnitudes of template flexibility did not affect performance of the adaptive process, because matching performance at the large magnitudes of μ—here μ ≥ 9—was crippled by spurious matches.)

To summarize, flexible matching yields much better performance than rigid matching at large disparity gradients, explained by the capability of flexible matching to identify corresponding image parts distorted due to the viewing geometry. Provided multiple degrees of flexibility, flexible matching is also capable of reliable performance at a much larger range of disparity gradients than rigid matching.

### Reduction of inter-ocular distortions by image blur

A method previously proposed to facilitate binocular matching and overcome inter-ocular distortions is to blur images. Blurring by the front-end (optical and post-optical) stages of the biological visual process (Campbell and Gubisch, 1966; Geisler, 1989) scatters luminance of monocular image features that do not align across the left and right images, thus improving inter-ocular registration of the features (e.g., Berg and Malik, 2001).

**Figure 9:**
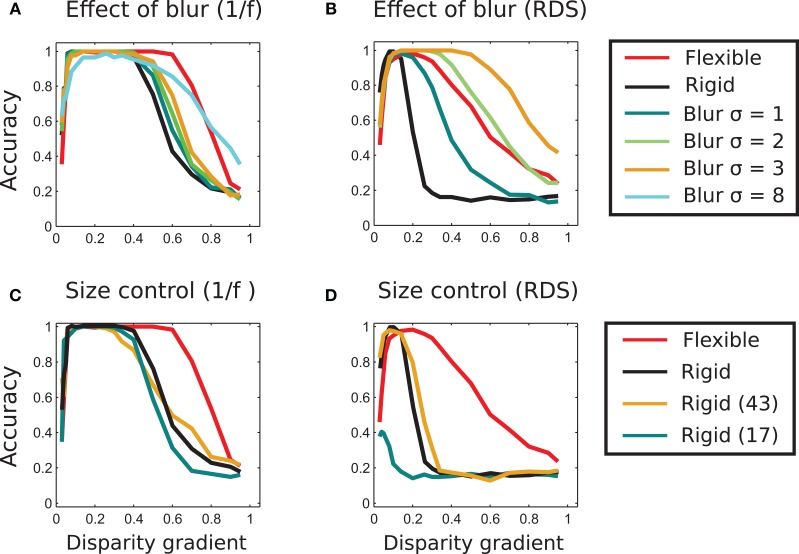
**Effect of blur and template size. (A)** Effect of image blur on matching performance in 1/f stereograms. The blue, green, and orange curves represent results of matching using different strengths of blur. The blurring marginally increases the range of perceived slants and performance of rigid matching. Even at very large blur (σ = 8 in the figure) the range of high performance is wide, but the maximal performance of unity is never reached. The curves representing performance of adaptive (red) and rigid (black) matching (using 33 × 33 pixel templates) are copied from Figure 8A for reference. **(B)** Effect of blur in random-dot stereograms. Here, the blurring significantly improves performance of the rigid method. For σ = 2 and 3, the range of slants for correctly identified tilts is wider than in the adaptive-flexible approach (red curve, as in Figure 8B.) Result for the blur of σ = 8 is not shown here to avoid clatter, as performance of rigid matching with the blur of σ = 3 already exceeds performance of flexible matching. **(C)** Effect of template size in rigid matching with 1/f stimuli. Rigid matching using templates smaller (17 × 17 pixels, blue curve) and larger (45 × 45, orange) than the original size (33 × 33, black) yielded approximately the same performance as the templates used in the rest of the study. **(D)** Effect of template size in rigid matching with random-dot stimuli. Performance of the larger template size (45 × 45 pixels, orange) is approximately the same as performance of the original size (Figure 7B). Performance is significantly reduced for smaller templates (17 × 17 pixels, blue). The curves representing performance of adaptive (red) and rigid (black) matching with the 33 × 33 pixels templates are copied from Figure 8B.

We applied Gaussian horizontal blur to each stimulus image of our stimuli. In Figures 9A,B we plot tilt discrimination performance using different amounts of blur, parameterized by size σ of the blurring kernel, for 1/f stimuli in panel A and random-dot stimuli in panel B. For 1/f stimuli, blur marginally improved performance of rigid matching, using σ ∈ [1 2 3]. (Results of rigid and flexible matching without blur are also shown, using the same black and red curves as in Figure 8). Increasing σ further reduced the peak performance of rigid matching, such that it failed to reach accuracy of 1 (shown for σ = 8 in panel A).

For random-dot stimuli, however, blur significantly improved performance of rigid matching, yielding better results than flexible matching. That is, advantages of flexible matching hold for the naturalistic stimuli and not for the random-dot stimuli.

### Role of template size

We ruled out the possibility that the better performance of flexible matching can be accounted for by a particular choice of template size. We did so by evaluating performance of a rigid matching procedure with template sizes 17 × 17 and 43 × 43 pixels (original size: 33 × 33 pixels). The results are plotted in Figure 9: for 1/f stimuli in panel C and for random-dot stimuli in panel D. The plots indicate that flexible matching (red curve, also shown in Figure 8A) performs significantly better than rigid matching with the other template sizes. We also plot performance of rigid matching using the (original) template size of 33 × 33 pixels (black curve, for comparison). Performance of the flexible model for template sizes 17 × 17 and 43 × 43 pixels (not shown in this figure to avoid clutter) was similar to performance of the adaptive procedure with template size 33 × 33 used in Experiment 1. Notably, performance of rigid matching is worse than that of flexible matching when the size of rigid templates is the same as the size of sub-templates of flexible matching (Figures 9C,D).

### Effect of the number of sub-templates

We repeated the above experiments using a larger numbers of sub-templates: nine and 16, using respectively 3 × 3 and 4 × 4 square sub-templates, 11 pixels wide for nine sub-templates and 9 pixels wide for 16 sub-templates. (Sub-templates slightly overlapped in the latter case since the 33-pixel templates did not evenly divide to the 9-pixel sub-templates.)

**Figure 10:**
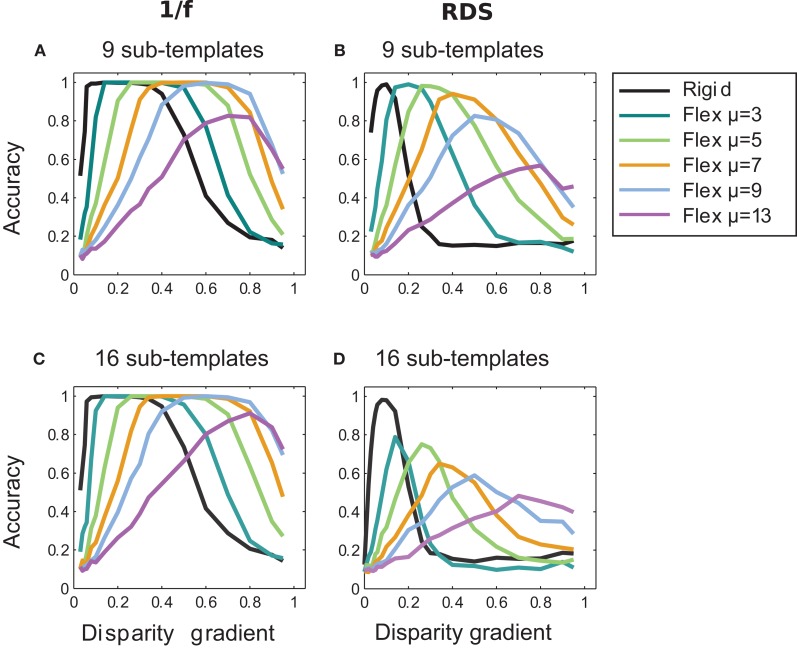
**Effect of the number of sub-templates.** Performance curves for different numbers of sub-templates with fixed magnitudes of μ: nine sub-templates in panels **A** and **B**, and sixteen sub-templates in panels **C** and **D**; for 1/f stimuli in **A** and **C**, and for random-dot stimuli in B and D. In 1/f stimuli, increasing the number of sub-templates improved performance for large magnitudes of μ (*cf.* Figure 7A where results are shown for four sub-templates). In random-dot stimuli, accuracy improved for nine sub-templates but it dropped for sixteen sub-templates (*cf.* Figure 7B).

Results of matching with the larger number of sub-templates for fixed μ are shown in Figure 10 for random-dot and 1/f stimuli. In comparison to results for the four sub-templates (Figure 7), the larger number of sub-templates improved performance at high magnitudes of μ (9 and 13) in the 1/f stimuli (Figures 10A and C), consistent with the view that the increased flexibility of matching has a larger tolerance to inter-ocular distortions. In the random-dot stimuli, performance improved for nine sub-templates but did not improve for sixteen sub-templates (Figures 10B and D), indicating that for the scarce luminance distribution in the random-dot stimuli, the additional flexibility of matching was beneficial up to a point at which the smaller sub-templates failed to capture patterns of luminance sufficiently unique to support reliable matching.

**Figure 11:**
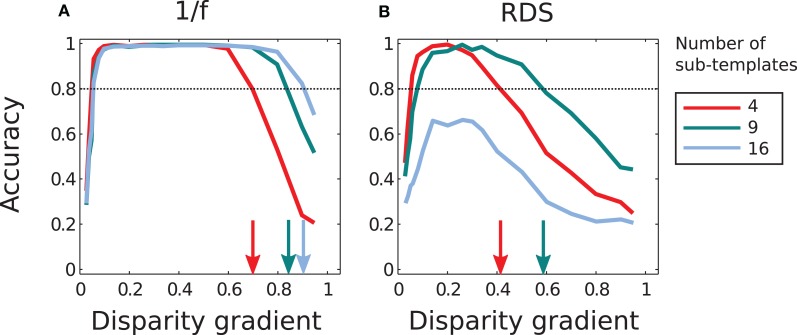
**Performance of the adaptive computation. (A)** In 1/f stimuli, the range of disparity-gradients for which performance was good increased as a function of the number of sub-templates. **(B)** In random-dot stimuli, performance improved for nine sub-templates, but further increase in the number of sub-templates impaired performance. The arrows mark the magnitudes of disparity gradient at which the descending arms of performance curves crossed the 0.8 level of accuracy. The red curves are the same as in Figure 8.

Figure 11 summarizes performance of the adaptive system that employs different numbers of sub-templates. Increasing the number of sub-templates improved performance, in particular for 1/f stimuli. (Now all magnitudes of μ were used in the adaptive computation since performance improved at large μ with nine and sixteen sub-templates, in contrast to the lack of such improvement with four sub-templates.) The range of disparity gradients at which performance was high increased with the number of sub-templates in the 1/f stimuli (panel A). But in random-dot stimuli performance improved with nine sub-templates while it was impaired with sixteen sub-templates, as explained in the previous paragraph (Figure 11A).

### Experiment 2

In Experiment 2 we investigate the ability of flexible matching to tolerate different amounts of inter-ocular distortion in different parts of the stimulus. Now we used a complex stimulus that contains multiple slants (Figure 6). We applied rigid and flexible matching procedures at all locations in this stimulus yielding maps of estimated tilt. Flexible matching employed four sub-templates. Instead of the hexagonal sampling used in Experiment 1, now positions of the sampling points were randomized (or else the regular placement of sampling points created artifacts in maps of estimated tilt) while care was taken that the arrangement of sampling points did not introduce a directional bias (i.e., that the covariance matrix of sample-point coordinates was proportional to the identity matrix and so Equation 8 held).

**Figure 12:**
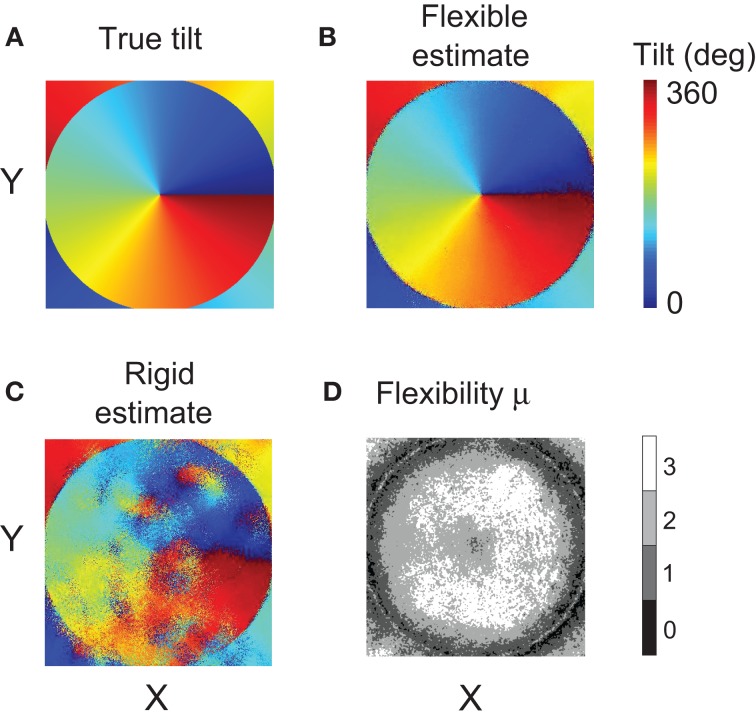
**Results of Experiment 2. (A)** Map of tilts in the stimulus. **(B)** Tilt map reconstructed using the flexible matching procedure with variable template flexibility. **(C)** Tilt map reconstructed using the rigid matching procedure. **(D)** Map of the magnitudes of μ selected by the flexible matching procedure at each stimulus location. (For clarity, the map was smoothened using a Gaussian kernel of size 5 × 5 pixels and of standard deviation equal to 1 pixel).

Figure 12 presents the map of true tilt in panel A, and the maps computed using different matching methods in panels B and C. Visual inspection of the maps makes it clear that flexible matching yielded a consistently more accurate tilt estimation than rigid matching. In particular, rigid matching performed poorly where the disparity gradient was large: on the flanks of the central peak of disparity. The tilt map by flexible matching is significantly more similar to the map of true tilt.

In Figure 12D we plot the magnitudes of template flexibility μ selected by the flexible matching procedure with variable template flexibility at each location in the stimuli. The plot shows that high magnitudes of μ were preferred where the disparity gradient was high (on the flanks of the disparity peak) and low magnitudes of μ were preferred where the gradient was low. The light ring in the periphery corresponds to the trough of disparity, where disparity gradient was zero and surface tilt was undefined. At these points, no particular magnitude of μ was preferred.

We computed mean errors of tilt estimated using the different matching methods: rigid, flexible with fixed magnitudes of μ, and flexible with variable magnitudes of μ. The mean error of tilt estimation was the mean absolute difference of the estimated and true tilts, modulo 180°, across all stimulus pixels. The mean error was below 5° for flexible matching, and it was larger than 30° for rigid matching.

## Discussion

We investigated how the well-known capacity of binocular complex cells for spatially invariant computation may benefit stereoscopic vision. We compared two approaches to binocular matching. One approach uses computations implicit in the standard model of binocular matching. We call this approach “rigid matching” because it favors identical left and right images. The other approach uses spatially invariant computations. It is “flexible” in the sense it allows for small independent displacements of fragments of left and right image parts, locally warping the images, thus helping to find corresponding image parts distorted by binocular projection. We modeled flexible matching using the computational framework of MAX-pooling (Riesenhuber and Poggio, 1999; Ullman et al., 2002; Serre et al., 2007a,b; Ullman, 2007).

Differences of outcomes from rigid and flexible matching were striking. Flexible matching was able to support efficient matching for a much larger range of slants than rigid matching, both in random-dot stereograms and in stimuli with naturalistic (1/f) luminance distributions (Figure 8). We found that performance of rigid matching significantly improved when combined with image blur (Berg and Malik, 2001) (our Figures 9A,B), but this result held only in random-dot stimuli. In stimuli with naturalistic luminance distributions, blurring did not improve performance of rigid matching, indicating that the spatially invariant computation is suited for perception of the natural visual environment.

In flexible matching, the amount of inter-ocular distortion tolerated by the matching process depends on the parameter we called template flexibility (μ, Equation 4) which represents different receptive field sizes of binocular complex cells. We showed that the amount of template flexibility most suitable for the current stimulus could be determined automatically, by WTA competition between cells with respective fields of different sizes. This competition may proceed concurrently and independently at many different stimulus locations, making binocular matching highly adaptive to the diverse scene geometry (Figure 12). It is possible that adaptive blurring can further improve performance: further studies should explore how adaptive blurring and adaptive flexible matching can be combined optimally.

Tanabe et al. (2004) found evidence of competition between hypotheses about binocular correspondence in cortical area V4. Such competition is akin to the process of “voting” in our study, which insured that the most suitable amount of matching flexibility was used at every location in the stimulus. Yet physiological studies have shown that the mechanisms that encode surface shape span many cortical areas from primary to inferotemporal cortical areas (Burkhalter and Essen, 1986; Uka et al., 2000, 2005; Qiu and von der Heydt, 2005; Sanada and Ohzawa, 2006), making it difficult to localize the neural substrate for these mechanisms. Indeed, it is likely that these mechanisms are distributed across several cortical areas.

We have focused on one component of binocular matching: the computation of inter-ocular similarity. We have shown that spatially-invariant computation of similarity is useful for discovering the corresponding image parts distorted by binocular projection. Since spatially-invariant computation is believed to be performed by binocular complex cells, we consider implications of our study for understanding the role of these cells in biological stereopsis.

The standard view is that binocular complex cells play the role of “disparity detectors”—i.e., they compute binocular disparity (Qiang, 1994; Ohzawa, 1998; Anzai et al., 1999). Our study suggests a different picture, that binocular complex cells cooperate in the computation of inter-ocular similarity. Indeed, receptive fields of individual complex cells are often too small to sufficiently represent the spatial-frequency content of the stimulus, which is essential for identifying corresponding image parts (as Banks et al., 2004, pointed out). We propose that inter-ocular similarity is computed by populations of complex cells with retinotopically adjacent respective fields of different sizes. This arrangement will have sufficient flexibility for finding corresponding image parts of variable size and under variable amount of image distortion.

Our results also suggest that binocular visual systems may do well by avoiding an early commitment to binocular disparity. Models of stereopsis commonly derive a single map of binocular disparity as soon as inter-ocular similarities are computed. In our framework, multiple disparity maps are computed using different magnitudes of template flexibility, simulating computations by binocular complex cells with receptive fields of different size. The alternative disparity maps coexist up to the stage where a higher-order stimulus property (such as tilt) is computed, taking advantage of the information that would be lost had the system committed to a single map of disparity early on. Computational studies of other sensory processes showed that preserving ambiguity about stimulus parameters until late stages of the sensory process can benefit system performance: in models of feedforward computations (e.g., Serre et al., 2007a,b and as implemented here) and also in models that involve feedback (e.g., Epshtein et al., 2008), where outcomes of computations at a late stage help to disambiguate results of early computations.

Our results indicate that the choice of stimulus for probing the computation of inter-ocular similarity is significant. Spatially invariant computations were more beneficial for stimuli with naturalistic distribution of luminance than for random-dot stimuli. The advantage was more pronounced as the flexibility of matching increased, both in terms of the spatial range of inter-ocular comparisons (Figures 7, 8) and in terms of the number of sub-templates (e.g., Figure 11). A likely reason for the stimulus effect is the fact that correlation measures of image similarity are highly sensitive to statistics of luminance in the images (Sharpee et al., 2006; Vidal-Naquet and Tanifuji, 2007). These findings suggest that results of studies of biological stereopsis that involved random-dot luminance patterns may need to be revisited. Also, the possibility should be considered that matching is adaptive and so changes in luminance statistics may yield a different outcomes of matching.

For example, Allenmark and Read (2010) found that rigid matching failed to account for human perception of slanted surfaces in random-dot stimuli. Allenmark and Read (2011) proposed that the inconsistency between outcomes of rigid matching and human performance could be resolved by adaptively increasing the size of the correlation window: the larger the disparity the larger the window (*cf.* Kanade and Okutomi, 1994). Future studies should compare human performance and performance of the alternative methods of matching using stimuli with naturalistic distribution of luminance. Moreover, a combination of the adaptive use of spatial invariance (as in our study) and adaptive use of the size of correlation window (as in Kanade and Okutomi, 1994 and Allenmark and Read, 2011) is likely to be most beneficial, such that a full model of the biological computation of inter-ocular similarity will incorporate adaptive spatially-invariant matching on multiple spatial scales, helping to explain the fact that biological vision is capable of reliable performance at yet higher disparity gradients (Tyler, 1973; Burt and Julesz, 1980; Allenmark and Read, 2010, 2011) than observed in the present study.

### Conflict of interest statement

The authors declare that the research was conducted in the absence of any commercial or financial relationships that could be construed as a potential conflict of interest.
